# Choosing increases the value of non-instrumental information

**DOI:** 10.1038/s41598-021-88031-y

**Published:** 2021-04-22

**Authors:** Matthew Jiwa, Patrick S. Cooper, Trevor T.-J. Chong, Stefan Bode

**Affiliations:** 1 School of Psychological Sciences, University of Melbourne, Melbourne, 3010 Australia; 2 Turner Institute for Brain and Mental Health, Monash University, Melbourne, 3800 Australia; 3 Alfred Health, Department of Neurology, Melbourne, 3004 Australia; 4 Department of Clinical Neurosciences, St Vincent’s Hospital, Melbourne, 3065 Australia

**Keywords:** Psychology, Human behaviour

## Abstract

Curiosity pervades all aspects of human behaviour and decision-making. Recent research indicates that the value of information is determined by its propensity to reduce uncertainty, and the hedonic value of the outcomes it predicts. Previous findings also indicate a preference for options that are freely chosen, compared to equivalently valued alternatives that are externally assigned. Here, we asked whether the value of information also varies as a function of self- or externally-imposed choices. Participants rated their preference for information that followed either a self-chosen decision, or an externally imposed condition. Our results showed that choosing a lottery significantly increased the subjective value of information about the outcome. Computational modelling indicated that this change in information-seeking behaviour was not due to changes in the subjective probability of winning, but instead reflected an independent effect of choosing on the value of resolving uncertainty. These results demonstrate that agency over a prospect is an important source of information value.

## Introduction

Individuals are required to choose whether to receive information about a vast range of topics. From the caloric contents of our favourite snacks, to the misdeeds of unknown celebrities, and our own genetic make-up, we now have more information available at the touch of a button—or swipe of a credit card—than ever before. Therefore, how we decide which information to view or avoid is of increasing personal, social, and commercial relevance.

Although information can be used to increase the likelihood or magnitude of future rewards, recent findings suggest that the value of information is not determined by this utility alone. Both human and non-human animals demonstrate a willingness to exchange rewards for information that cannot be used to influence the probability or magnitude of future rewards^1–7^. Recent neurophysiological data have shown that such non-instrumental information is encoded by similar neural circuits as primary reinforcers^5,8–10^, with unexpectedly informative signals producing similar neural responses to unexpectedly positive outcomes^1,8^. Beyond its instrumental value (the extent to which that information can be used to increase the magnitude or likelihood of future rewards), the subjective value of information is further determined by both its hedonic value (the expected affective response to the information), and its cognitive value (the degree of uncertainty that viewing the information is expected to resolve)^5,10–12^.

Importantly, however, intrinsic biases and contextual factors may affect the contributions that each of these factors make to the overall value of an informational prospect^11^. The hedonic value of information may be particularly susceptible to bias. Typically, it is found that prospects that offer a greater chance of a positive outcome (or lesser chance of a negative one) elicit a greater willingness to pay to view that information than those with a more negative outlook^5,12,13^. However, as findings in the fields of behavioural economics and cognitive psychology have repeatedly shown, individuals’ predictions of outcomes are often open to systematic biases and heuristics that may, in turn, affect the value of information pertaining to those prospects^11,14,15^. A common example is choice-induced preference change—where the act of choosing an option increases its subjective value relative to alternatives both during and after the decision making process^16–23^. In addition, choice may not only increase an the subjective value of a chosen option, but also an individual’s curiosity about it^24^. However, the evidence suggesting this does not account for pre-choice preferences, nor can it comment on whether increases in curiosity are due to choice-induced modulation, or more indirectly through an increase in the subjective value of the chosen information.

Critically, individuals predict more favourable outcomes for prospects over which they have agency, rather than those that are assigned to them—a phenomenon termed the “illusion of control” (IOC)^14,25,26^. As the probability of outcomes is crucial to the valuation of prospects, with higher probability of favourable outcomes leading to a higher valuation of a prospect^27–29^, there are clear theoretical implications of this subjective change for both the behavioural and neural components of decision-making. As the neural responses to rewards are typically encoded as the signed difference between an actual reward and an expected reward (reward prediction error; RPE), we may expect that an increased subjective probability of positive outcomes would lead to an attenuated RPE in the event of a positive outcome^30^. However, evidence suggests that neural responses in the striatum may not be affected by the IOC^31^. This finding suggests that the probability of winning used in the computation of the RPE may differ from the probability of winning reported by participants^31,32^.

The question of whether subjective information value is affected by biases in probability produced by the IOC remains an open one. In this study, we aimed to test whether agency over an arbitrary choice between alternative lotteries with identical probabilities of winning systematically increased: (a) participants’ confidence in a winning outcome (measured through self-reported confidence levels) and (b) the valuation of receiving early, non-instrumental information about the outcome of that prospect (measured by participants’ willingness to exchange rewards for the early knowledge of a lottery’s outcome). In addition, we used computational modelling to test whether the potential increase in confidence in winning the lottery could explain any observed increase in the information value, or if the increase in information valuation was otherwise better explained by a distinct effect of agency on the factors determining the subjective value of information.

## Results

**Figure 1:**
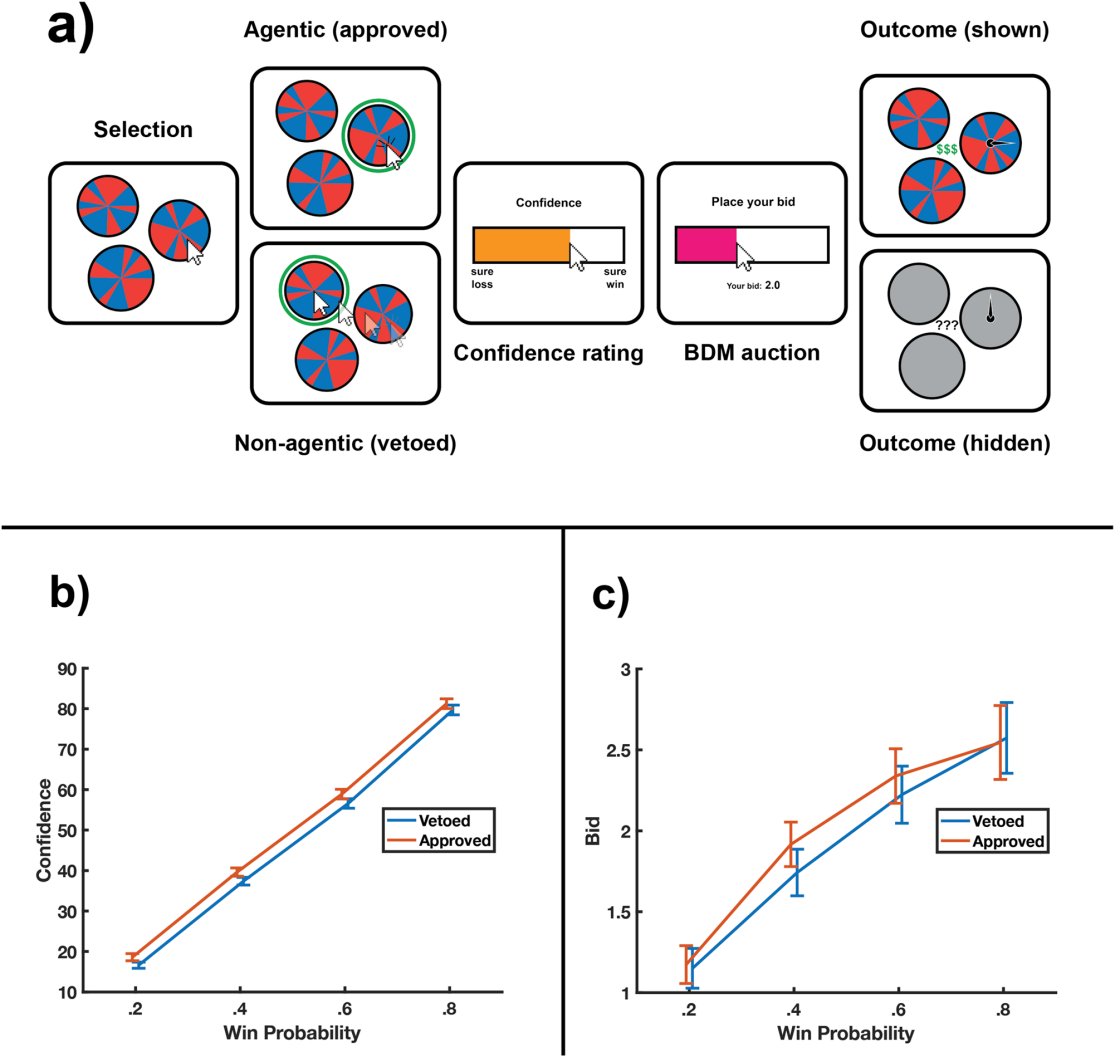
Experimental design and results. (**a**) On each trial, participants made a selection between three equivalent roulette wheels. Their choice was either approved or vetoed, in which case the cursor was moved to and selected an alternative option. They then rated their confidence to win that trial and submitted a bid in a Becker–DeGroot–Marschak (BDM) auction to determine whether the outcome would be revealed or hidden. If their bid was successful, the outcome was shown, otherwise, it was hidden. (**b**) Mean confidence ratings (on a scale from 0 = “sure loss” to 100 = “sure win”) for each possible probability of winning. Confidence ratings were significantly higher when participants’ selection of lottery was approved. Error bars represent the standard error of the mean. (**c**) Mean bid size in points for each possible probability of winning. Bids were significantly larger when participants’ selection of lottery was approved. Error bars represent the standard error of the mean.

To assess the influence of choosing on information valuation, we manipulated participants’ perceived agency during a simple lottery, in which the decision to play a specific “roulette wheel” could be approved or vetoed. In a series of trials, participants were presented with three roulette wheels, which they were accurately instructed each had the same probability of winning, and asked to choose their preferred prospect, similar to Kool et al.^31^ (see Fig. 1a). The participant’s selection was either approved (granting agency over the trial) or vetoed (removing agency from the trial). These lotteries utilised scrambled roulette wheels comprising segments of a winning colour and a non-winning colour. The three wheels on each trial were rotated versions of the same configuration of otherwise identical colour segments, with the probability to win 0.2, 0.4, 0.6 or 0.8 (see “Methods” for details).

Following the approval/veto stage, we probed the effects of agency on information value using two methods. First, participants rated their confidence to win that trial using a continuous scale. Second, participants were administered a Becker–DeGroot–Marschak (BDM) auction^33^ in which they stated the maximum cost they would be willing to incur in order to reveal the outcome of the lottery immediately. Their bid was then compared to a random bid made by the computer, and, if the participant’s bid was higher than the computer’s bid, the latter would be deducted from the participant’s points total, and they would learn the outcome of the trial. If their bid was lower, they were instructed that the lottery would still be played out and winnings allocated; however, they would not learn the outcome immediately. The magnitude of the participant’s bid therefore served to represent the maximum value they would be willing to pay in order to view the non-instrumental information about that trial’s outcome. This auction procedure guaranteed that the most realistic valuation of the non-instrumental information was obtained on each trial.

To characterise participants’ preferences for acquiring this information, we also constructed a series of computational models which were fit to the participants’ trial-wise bidding patterns. These models included a null model, which assumed no effect of agency on information-seeking behaviour, as well as a series of models that characterised differences in information valuation across agentic and non-agentic trials as due to changes in the subjective probability of winning, the subjective value of resolving uncertainty, the subjective value of anticipating a positive outcome, or simply to the desirability of information.

### Agency increases win expectancy and information value

To assess whether participants’ subjective probability of a positive outcome was affected by their agency over the lottery, their confidence ratings were compared across agentic and non-agentic trials. A $2 \times 4$ repeated measures analysis of variance (ANOVA) with within-subjects factors of agency (agentic or non-agentic) and win probability (0.2, 0.4, 0.6 or 0.8) indicated that confidence in positive outcomes was modulated both by the probability of winning, $F(3, 114) = 949.90, p< 0.001$ and possession of agency, $F(1, 38) = 30.78, p < 0.001$. As shown in Fig. 1b, confidence ratings were an average of 2.04% higher when participants had agency over which lottery they played, indicating that this paradigm successfully elicited the IOC and replicated earlier findings^14,31^.

Next, we examined the influence of agency on the perceived value of information by assessing participants’ willingness to pay for non-instrumental information. A $2 \times 4$ repeated measures ANOVA with within-subjects factors of agency (agentic or non-agentic) and win probability (0.2, 0.4, 0.6 or 0.8) indicated that participants’ bid magnitude was positively predicted by both win-probability, $F(3, 114) = 49.92, p < 0.001$ and agency, $F(1, 38) = 14.27, p < 0.001$, see Fig. 1c. There was also a significant interaction effect between the win-probability and agency, $F(3, 114) = 4.41, p = 0.006$. A post-hoc comparison of bid-magnitude across probabilities revealed that the difference between agentic and non-agentic bids was only significant for trials in which the probability of winning was 0.4, $t(38) = 3.96, p = 0.001$ or 0.6, $t(38) = 2.92, p = 0.023$ after Bonferroni corrections were applied.

These findings suggest that participants were willing to sacrifice a significantly higher proportion of their winnings in order to learn the outcome of a trial if they had agency over the selection of the prospect (compared to when they did not have agency), with the strongest effect found in scenarios with relatively higher uncertainty.

### Agency increases the value of resolving uncertainty

To assess whether the increase in information desirability could be explained by the increase in the subjective probability of a positive outcome, we constructed five computational models. The first model assumed no effect of agency on information preference. Based on previous findings, this *null model* instead constructed predictions only from a weighted combination of the win-probability, the uncertainty of the prospect, and a subject-specific constant (see “Methods”).

We also constructed a series of alternative models to assess different characterisations of the contributions of agency to information value. In the *probability-shifted model*, shifts in the subjective probability of winning across agentic and non-agentic trials were permitted, such that the probability of winning on agentic trials was shifted by the magnitude of an additional subject-specific free parameter.

In addition, we constructed three further models to test for changes in the subjective valuation of each constituent parameter of the null model. These models use an additional free parameter to allow the contributions of reward probability (*agency reward model*), uncertainty (*agency uncertainty model*), and the constant value of information (*agency constant model*) to vary across agentic and non-agentic trials.

To compare model fits, we used the Watanabe-Akaike Information Criterion (WAIC) measure of out-of-sample prediction error^34,35^. WAIC calculation involves the subtraction of a measure of model complexity from a goodness of fit measure. It was chosen over other information criteria (e.g., the Deviance Information Criterion) as it has a higher power rate and does not assume the posterior distribution to be Gaussian^36,37^. The WAIC suggests that the *agency uncertainty model* is preferred, with the *null model* underperforming compared to each of the other models, suggesting that differences in curiosity between chosen and non-chosen lotteries were best accounted for by an increase in the value of resolving uncertainty, rather than due to the shift in the subjective probability of a positive outcome (see Table 1). Further, the probability-shift parameter of the *probability-shifted model* demonstrated poor adherence to the reported increase in subjective win probability, as shown by their weak correlation, $r(38) = -0.10$. Posterior predictive checks demonstrated an excellent fit of the *agency uncertainty model* to the data (Supplementary Fig. S1). The final parameter estimates for the *agency uncertainty model* are shown in Supplementary Fig. S2.

**Table 1:** Watanabe-Akaike Information Criterion (WAIC) for each of the discussed models. A smaller value indicates a better fit to the data. The WAIC for the best fitting model is highlighted in bold. The difference from the best-fitting model is represented as $\Delta$WAIC.

Model	Free parameters (per participant)	WAIC	$\Delta$WAIC (S.E.M)
Null	4	8065.11	31.60 (11.54)
Probability-shifted	5	8040.51	7.00 (14.48)
Agency reward	5	8039.78	6.27 (15.29)
Agency uncertainty	5	**8033.51**	–
Agency constant	5	8038.90	5.39 (14.40)

Together, these analyses show that subjective information value was increased in agentic, relative to non-agentic contexts, particularly when uncertainty was maximal. Computational modelling contradicts the notion that the increased value of information is simply an effect of the increase in the subjective probability of a positive outcome for agentic prospects. Instead, it suggests that the value of resolving uncertainty is increased in situations involving agency.

## Discussion

In this study, we assessed how choosing a prospect affects information valuation. Each participant was given the opportunity to bid money in exchange for immediate, but entirely non-instrumental, information about the outcomes of lotteries. On each trial, the participant could possess agency over which lottery would determine their winnings, or the lottery could be randomly assigned. First, we replicated earlier findings that agency over choosing one’s lottery increased the perceived confidence in a positive outcome of the lottery^31^. Consistent with previous findings, we also demonstrated that participants showed a preference for information pertaining to prospects with high likelihood of revealing positive outcomes, as well as those with high uncertainty^5,10,12^. Our results further showed that participants were willing to place higher bids in order to learn the outcome of a trial over which they had agency, suggesting that they valued the information more under those circumstances. This was particularly so for trials with a higher degree of uncertainty about the outcome. Computational modelling analyses indicated that the agency-related subjective change in the probability of a positive outcome did not provide the best account for the increase in information valuation for agentic choices. The results were best explained through an increase in the subjective value of resolving uncertainty for agentic prospects. These findings are not directly explained by existing theories of information-seeking behaviour^11^.

The success of the *agency uncertainty model* over the other competing models may be explained by a tight, automatic association between agency and the cognitive value of information. The cognitive value of an information signal is determined by the extent to which that information signal is able to reduce the uncertainty surrounding one’s own mental model of the world around them^11^. Outcomes of events produced through agentic means may be perceived as possessing greater cognitive value for two primary reasons. First, they often inform us of the outcomes of our own decisions or actions, and therefore may affect our mental model of concepts related to our self-perception, including our attitudes towards ourselves and towards other concepts^38^. More generally, experiencing the outcomes of our actions may allow us to assess the accuracy of our existing predictions of action-outcome contingencies present in our mental models by either validating or challenging these predictions. Second, outcomes of our own actions may be associated with a higher cognitive value simply because we are typically more likely to act upon or interact with objects or concepts that have a greater relevance to us (and, therefore, have a more significant presence in our mental model of the world and elicit more curiosity). In support of this, research has demonstrated that information on topics that are selected by an individual lead to higher levels of curiosity than those that are randomly selected^24^. Of course, participants in the current experiment could not expect to extract more cognitive value from information about lotteries under agentic conditions. However, the increased valuation of information arising from agentic decisions may be attributed to the association between agency and cognitive value being “overlearned”; learned beyond the point of automaticity such that it is applied dogmatically. The phenomenon of overlearning is typically associated with improvements in memory retention^39^, but can also strengthen stimulus-response associations^40^. If this association between agency and cognitive value were to be the subject of overlearning, this may result in the higher subjective valuation of agentic outcomes observed in the present study.

Alternatively, the current findings could be explained by an overlearning of the perceived contingency between agency and the utility of information. Under normal, everyday circumstances, information about decision outcomes can typically be used to inform future decisions^41^. Conversely, information learned in an environment in which one does not possess agency may not be perceived as useful, because we cannot use such information to inform future decisions. Though the association between agency and utility was not present in the current experimental framework, an overlearning of this association may account for the relationship between agency and the subjective value of resolving uncertainty.

Equally, the failure of the other competing models highlight key shortcomings in the ability of existing accounts to explain the present findings. For example, choice-induced preference change^16–24^ alone cannot account for the present findings, as it predicts that the value of information should increase uniformly across agentic prospects (consistent with the *agency constant model*), or that the value of information should increase due to the indirect effects of an increase in the subjective value of the associated rewards (consistent with the *probability-shifted model*). The underperformance of these models relative to the *agency uncertainty model* indicates that we cannot attribute the present results to choice-induced preference change alone.

Other contemporary accounts argue that agency provides an opportunity for self-enhancement (the motivation to view oneself in a positive manner), which could supplement information value. Previous studies have shown that the delivery of outcomes from agentic decisions activates neural circuits that process self-referential information^31,42–48^. However, information-seeking with the goal of self-enhancement typically involves an active search for flattering (positively-valenced) information^49,50^. In the current experimental framework, this should manifest in an increase in the perceived value of information pertaining to agentic prospects with a high probability of winning, as conceptualised by the *agency reward model*. The poor fit of this model relative to that of the *agency uncertainty model* indicates that a self-enhancement explanation alone cannot account for these data.

It should be noted that our conclusions are somewhat limited by the small effect sizes. This highlights the comparative importance of the primary information-seeking drivers, such as the anticipation of positive outcomes. Further, in the present study, the agentic condition in which participants chose their own prospect was contrasted to a condition in which their choice was vetoed^31^. Arguably, the veto might have evoked cognitive processes beyond the experience of a lack of agency, for example the feeling of a loss of agency, or dissatisfaction with losing control in general. Future studies could consider a truly passive control condition in which no agency exists in the first place; however, this might come with the danger of task disengagement. These findings also leave open further questions about whether subjective changes in reward value and probability influence the value of non-instrumental information about those rewards. One example of this is effort discounting, in which the value of a reward is reduced when it is earned through effortful, as opposed to non-effortful means^51–55^. While evidence for the IOC has not been demonstrated in neural indices of reward^31^, the modulation of reward signals has been demonstrated in tasks requiring effort^53,54^. Investigating information-seeking behaviour in this context would provide insight into whether the findings of the present study are unique to the IOC, or whether they can be generalised to other, neurally observable subjective alterations to the expected value of a prospect.

Finally, though each of the alternative models outperformed the null model, this is likely because each is able to make predictions that mimic the performance of the *agency uncertainty model*. This is exemplified by the poor correlation between the probability-shift parameter of the *probability-shifted model* and the subjective increase in win-probability reported by participants. As such, though the success of each alternative model relative to the null model would promote the conception of hybrid models that include combinations of the agency-modulated parameters, the inclusion of combinations of these parameters would constitute redundancy. Consequently, models that include more than one agency-modulated parameter produce divergent model fits, as very different combinations of the parameters lead to similar model performance, making model identification difficult and inferences about such models unreliable.

In sum, the findings of the present study demonstrated an increase in the perceived value of resolving uncertainty about the post-decisional outcomes of prospects when those prospects are selected through agentic means, as opposed to when agency is removed. This increase in value was not explained by a change in the subjective probability of a positive outcome. Instead, it may be attributed to an overlearning of the association between choice and the instrumentality or cognitive value of information.

## Methods

### Participants

Forty-seven participants (30 female, 17 male, $$M = 23.47, SD = 2.69$$) completed the experiment. Four were excluded from analyses for failing to meet the pre-determined accuracy threshold for confidence ratings of $$\pm 20$$% from the true probability across all four probability levels, indicating an insufficient understanding of the real probability of winning. A further four were excluded for failing to show any evidence of information valuation, suggesting a general lack of interest in the lottery’s outcome. The remaining sample of 39 participants (25 female, 14 male) were aged between 18 and 36 years of age (*M* = 23.21, *SD* = 2.65). Participants received a reimbursement of AUD $15 for their participation, and were instructed that they could win an additional reward of up to $5 available depending on the results of the experiment. However, at the end of the experiment, all participants were awarded the full $20 reimbursement, regardless of performance. Informed consent was provided by all participants, and research was conducted in accordance with the Declaration of Helsinki. All study protocols were approved by The University of Melbourne Human Research Ethics Committee (ID 1954969).

### Procedure

All stimuli were presented using the Psychophysics Toolbox^56^ running on MATLAB R2018a (The Mathworks, Natick, MA) on a cathode-ray tube (CRT) monitor with a resolution of $1280 \times 1024$ pixels and a screen refresh rate of 60 Hz.

Before commencing with the experiment, participants were provided with written and verbal instructions for the task and were permitted to complete a series of practice trials (with a minimum of 20) until they felt confident to continue to the main task. Participants were instructed that, on each trial, they were to choose from three roulette wheels, each of which had an equal probability of producing a winning outcome. The roulette wheels comprising segments of red and blue, with the either the red or blue connoting a winning outcome (counter-balanced across participants). Importantly, participants were fully aware that there was no difference in the probability of winning for each of the wheels on each trial. They were instructed that a spinner was going to be rotated around one of the wheels and if it landed on their winning colour, they would win 50 points. Losses were worth 0 points, with their overall total points at the end of the experiment determining the size of the monetary payment at the end of the experiment. They were further told that on some trials, their choice of wheel would be “approved” while on other trials, the computer would “veto” their decision and select a different wheel.

The main experiment consisted of ten blocks, each containing twelve trials. In each block, three trials of each of the four win probabilities (0.2, 0.4, 0.6, 0.8) were presented. On each trial, three possible roulette wheels were initially shown. The wheels consisted of 40 segments of equal size. To construct the three roulette wheels, the win probability was multiplied by the total number of segments, and the resultant number of segments were shaded in the participant’s winning colour, with the remainder shaded in the alternate colour. The order of the segments was randomly shuffled. To construct the other two wheels, this roulette wheel was then duplicated, and rotated $120^{\circ }$ clockwise and counter-clockwise. This ensured that the wheels were structurally identical, such that differential segment distribution could not produce preferences based on perceived differences in win probability. Participants were instructed that each of the three lotteries were equivalent. During the post-experiment debrief, participants were also asked to describe their method of choosing a lottery, such that any participants who indicated they believed the lotteries were not equivalent could be excluded. Two participants indicated that they believed the lotteries had not been equivalent. Both also failed to meet the accuracy requirement for confidence ratings and were excluded.

On each trial, the centre-point of each wheel was positioned along the circumference of an imaginary circle centred at the mid-point of the screen. The first wheel was placed randomly along this circumference, with the remaining two positioned at $120^{\circ }$ and $240^{\circ }$ clockwise, respectively. The mouse was initially positioned at the centre of the screen. Participants were instructed to select one of the three options by clicking on it with the mouse within 2.5 s of stimulus onset. If the participant did not respond in time, they received the feedback, “too slow”, and the trial was restarted with different stimuli (with the same win probability).

If the participant’s selection was approved, their selected wheel was highlighted with a green circle. If their selection was vetoed, the cursor would flash, and was then automatically moved to one of the other two options by the computer. The newly computer-selected option was then highlighted with the green circle. Approved and vetoed selections were pseudo-randomised, such that each occurred on half of the trials in each block. The proportion of approved/vetoed trials was kept equal across all win probabilities, and it was ensured that equal numbers of approved and vetoed trials resulted in winning outcomes.

Following their selection, there was a 2 s delay, after which participants completed a confidence rating to indicate how likely they perceived a winning outcome to be^31^. This was completed by moving the mouse along a continuous scale with markings of “sure loss” and “sure win” at either end. Cursor position was randomised prior to the appearance of the scale on each trial. Participants were given 4 s to complete this rating, with the position of the mouse at the end of the 4 s period recorded as their confidence rating.

After this, participants completed a procedure that allowed us to assess the value they placed on receiving immediate information about the outcome of the trial. We used a Becker–DeGroot–Marschak (BDM) auction^33^ to determine whether they would learn the outcome of the trial or not. In this procedure, the position of the cursor was randomised before participants had 5 s to make a bid of between 0 and 5 points (with increments of 0.1). The size of their bid was then compared against a randomly generated “price” of between 0.1 and 5 points. If the participant’s bid was equal to or higher than this price, that price would be deducted from their score, and they were shown the outcome of the lottery at the end of the trial. Otherwise, no points were deducted, but the outcome of the lottery was kept secret. Participants were explicitly instructed that the outcome of the bid had no bearing on whether they won or lost on any given trial, only whether they would find out the outcome immediately. Any winnings were added to their total score. All bids were made by moving the mouse along a horizontal scale, with the position of the mouse at the end of the 5 s duration determining the size of the bid.

If their bid was successful (i.e., if it was greater than the price), a green bar flashed on the screen for 1 s. The initial three roulette wheels were then redisplayed, with a spinner placed on the (self- or computer-) selected wheel. The spinner rotated around the selected wheel for 2.5 s, stopping on either a winning or losing segment. If the spinner stopped on a winning segment, the feedback “$$$” was shown. If it stopped on a losing segment, the feedback “XXX” was shown.

If the participant’s bid was unsuccessful, a red bar flashed on the screen for 1 s. The wheels were not shown with their original segments, but were instead replaced by uniformly grey wheels. The spinner rotated around the wheel for 2.5 s, returning to the top of the wheel, and the feedback “???” was shown, indicating that the outcome of the trial remained unknown. Participants were explicitly instructed that, when the grey wheel was shown, the final position of the spinner in no way reflected its position on the selected wheel and was intended as a filler screen only.

Each trial was followed by an inter-trial interval of 0.4 s. Participants were provided with an un-timed break after each block of 12 trials.

### Computational models

We constructed a series of computational models to characterise the value that individuals placed on each lottery. All models were fit using Hamiltonian Monte Carlo sampling as implemented in Stan^57^. Each model was fit using four parallel chains with a warm-up period of 1500 samples each followed by 5000 samples drawn from the converged chains.

#### Null model

Based on the previously established findings in this area^5,10,12^, the *null model* operationalised the prediction that participants’ bids were dependent on: (1) the likelihood of a positive outcome, and (2) the uncertainty of the current prospect. Uncertainty was defined as the entropy of the prospect, with entropy calculated by the Shannon function^58^, see Eq. (1). To maintain uncorrelated model parameters, this entropy was centred by subtracting the mean level of entropy across all trials, as shown in Eq. (2).

1 $$\begin{aligned}H(X) = -\sum _{i=1}^{n} P(x_i) \log _{2} P(x_i) \end{aligned}$$

2 $$\begin{aligned}&{\bar{H}} = H(X) - \frac{H(0.2) + H(0.4) + H(0.6) + H(0.8)}{4} \end{aligned}$$

As a result, the null model predicts that the value of information is determined by the weighted, linear combination of win probability, uncertainty, and a constant to capture any other sources of information valuation (Eq. 3).

3 $$\begin{aligned} \mu _{V[i]} = \beta _{U[i]} \, {\bar{H}} + \beta _{W[i]} \, P(W) + \phi _{i} \end{aligned}$$

Here, the free parameter $\beta _{W}$ dictates the extent to which the probability of winning (*P*(*W*)) modulates the subjective value of information, with greater values of $\beta _{W}$ leading to greater increases in subjective information value with increasing win probability. Similarly, $\beta _{U}$ corresponds to the magnitude of change in the subjective value of information associated with the level of uncertainty on the given trial. Finally, the free parameter $\phi$ is a subject-specific constant that modulates the value of information uniformly across all trials.

To estimate this set of parameters, we employed a hierarchichal Bayesian estimation strategy that assumes that each subject’s parameters are drawn from a joint group-level distribution such that the parameters for subject *i* are constrained to being drawn from the prior distributions:

$$\begin{aligned}&\beta _{W} \sim {\text {Normal}}(\mu _{\beta _{W}}, \sigma _{\beta _{W}}) \\&\beta _{U} \sim {\text {Normal}}(\mu _{\beta _{U}}, \sigma _{\beta _{U}}) \\&\phi \sim {\text {Normal}}(\mu _{\phi }, \sigma _{\phi }) \end{aligned}$$

Each of these prior distributions was weakly informative, restricting the model to reasonable areas of the possible parameter space. The half-Cauchy distribution was used as a prior for standard deviations as it restricts away from large values while still offering some prior information^59^.

$$\begin{aligned} \mu _{\beta _{W}}, \mu _{\beta _{U}}, \mu _{\phi }&\sim {\text {Normal}}(0, 2) \\ \sigma _{\beta _{W}}, \sigma _{\beta _{U}}, \sigma _{\phi }&\sim {\text {HalfCauchy}}(0, 2) \end{aligned}$$

Finally, the outcome variable was also assigned weakly informative group-level hyperparameters and restricted to a truncated normal distribution with limits of 0 and 5, to match the lower and upper bid limits. The mean of this distribution, $\mu _{V[i]}$, was given by Eq. (3).

$$\begin{aligned} V_{i}&\sim {\text {TruncNormal}}(\mu _{V[i]}, \sigma _{V[i]}), \; \in [0, 5] \\ \sigma _{V}&\sim {\text {HalfCauchy}}(0, 2) \end{aligned}$$

#### Probability-shifted model

The *probability-shifted model* allowed for the adjustment of the subjective probability of a winning outcome depending on whether or not participants could choose the lottery, as expected under the illusion of control. This was achieved by allowing probability to be adjusted by an additional free parameter, $\beta _{prob}$, such that the probability of winning used in the calculation of entropy and information valuation was given by Eq. (4). Here, *A* is a binary variable that equals 1 on trials in which the participant has agency (i.e., when their selection is approved) and 0 when the participant does not have agency (i.e., when their selection is vetoed).

4 $$\begin{aligned} P_s(W) = P(W) + \beta _{prob} \, A(t) \end{aligned}$$

The entropy equation used to calculate uncertainty was also updated to employ the use of the shifted probability such that it was calculated using Eqs. 5 and 6.

5 $$\begin{aligned}H_s(X) = -\sum _{i=1}^{n} P_s(x_i) \log _{2} P_s(x_i) \end{aligned}$$

6 $$\begin{aligned}&{\bar{H}}_s = H_s(X) - \frac{H_s(0.2) + H_s(0.4) + H_s(0.6) + H_s(0.8)}{4} \end{aligned}$$

The distribution of the $\beta _{prob}$ parameter was truncated at $\pm 0.2$, to prevent the subjective probability of winning from exceeding 0 or 1. The model otherwise followed the same format as the *null model*. The full notation for the *probability-shifted model* is provided below:

$$\begin{aligned} V_i&\sim {\text {TruncNormal}}(\mu _{V[i]}, \sigma _{V[i]}), \; \in [0, 5] \\ \mu _{V[i]}&= \beta _{U[i]} \, {\bar{H}}_s + \beta _{W[i]} \, P_s(W) + \phi _{i} \\ \sigma _{V}&\sim {\text {HalfCauchy}}(0, 2) \\ \beta _{W}&\sim {\text {Normal}}(\mu _{\beta _{W}}, \sigma _{\beta _{W}}) \\ \beta _{U}&\sim {\text {Normal}}(\mu _{\beta _{U}}, \sigma _{\beta _{U}}) \\ \phi&\sim {\text {Normal}}(\mu _{\phi }, \sigma _{\phi }) \\ \beta _{prob}&\sim {\text {TruncNormal}}(\mu _{\beta _{prob}}, \sigma _{\beta _{prob}}), \; \in (-0.2, 0.2) \\ \mu _{\beta _{W}}, \mu _{\beta _{U}}, \mu _{\phi }&\sim {\text {Normal}}(0, 2) \\ \mu _{\beta _{prob}}&\sim {\text {Normal}}(0.02, 0.05) \\ \sigma _{\beta _{W}}, \sigma _{\beta _{U}}, \sigma _{\phi }&\sim {\text {HalfCauchy}}(0, 2) \\ \sigma _{\beta _{prob}}&\sim {\text {HalfCauchy}}(0, 0.05) \\ \end{aligned}$$

#### Agency modulation models

Finally, a series of models were constructed that allowed the magnitude of each of the factors used to compute the value of information in the null model to vary based on the presence or absence of agency. These models explored the possibility that agency modulates the value of information via a modulation of the value of the anticipation of a positive outcome, the resolution of uncertainty, or the constant underlying value of information.

The *agency reward model* predicts that the value of information in agentic contexts differs from that in non-agentic contexts due to a difference in the valuation of the anticipation of a positive outcome. This was achieved by adding the product of a binary parameter, *A*, and an additional free parameter, $\beta _{aW}$, to the existing free parameter $\beta _W$. The full notation for the *agency reward model* is provided below:

$$\begin{aligned} V_i&\sim {\text {TruncNormal}}(\mu _{V[i]}, \sigma _{V[i]}), \; \in [0, 5] \\ \mu _{V[i]}&= \beta _{U[i]} \, {\bar{H}} + (\beta _{W[i]} + \beta _{aW[i]} \, A(t)) \, P(W) + \phi _{i} \\ \sigma _{V}&\sim {\text {HalfCauchy}}(0, 2) \\ \beta _{W}&\sim {\text {Normal}}(\mu _{\beta _{W}}, \sigma _{\beta _{W}}) \\ \beta _{U}&\sim {\text {Normal}}(\mu _{\beta _{U}}, \sigma _{\beta _{U}}) \\ \phi&\sim {\text {Normal}}(\mu _{\phi }, \sigma _{\phi }) \\ \beta _{aW}&\sim {\text {Normal}}(\mu _{\beta _{aW}}, \sigma _{\beta _{aW}}) \\ \mu _{\beta _{W}}, \mu _{\beta _{U}}, \mu _{\phi }&\sim {\text {Normal}}(0, 2) \\ \mu _{\beta _{aW}}&\sim {\text {Normal}}(0, 1) \\ \sigma _{\beta _{W}}, \sigma _{\beta _{U}}, \sigma _{\phi }&\sim {\text {HalfCauchy}}(0, 2) \\ \sigma _{\beta _{aW}}&\sim {\text {HalfCauchy}}(0, 1) \\ \end{aligned}$$

Similarly, the *agency uncertainty model* predicts that agency affects information valuation due to a modulation of the value of resolving uncertainty for agentic prospects. This was achieved by adding the product of a binary parameter, *A*, and an additional free parameter, $\beta _{aU}$, to the existing free parameter $\beta _U$. The full notation for the *agency uncertainty model* is provided below:

$$\begin{aligned} V_i&\sim {\text {TruncNormal}}(\mu _{V[i]}, \sigma _{V[i]}), \; \in [0, 5] \\ \mu _{V[i]}&= (\beta _{U[i]} + \beta _{aU[i]} \, A(t)) \, {\bar{H}} + \beta _{W[i]} \, P(W) + \phi _{i} \\ \sigma _{V}&\sim {\text {HalfCauchy}}(0, 2) \\ \beta _{W}&\sim {\text {Normal}}(\mu _{\beta _{W}}, \sigma _{\beta _{W}}) \\ \beta _{U}&\sim {\text {Normal}}(\mu _{\beta _{U}}, \sigma _{\beta _{U}}) \\ \phi&\sim {\text {Normal}}(\mu _{\phi }, \sigma _{\phi }) \\ \beta _{aU}&\sim {\text {Normal}}(\mu _{\beta _{aU}}, \sigma _{\beta _{aU}}) \\ \mu _{\beta _{W}}, \mu _{\beta _{U}}, \mu _{\phi }&\sim {\text {Normal}}(0, 2) \\ \mu _{\beta _{aU}}&\sim {\text {Normal}}(0, 1) \\ \sigma _{\beta _{W}}, \sigma _{\beta _{U}}, \sigma _{\phi }&\sim {\text {HalfCauchy}}(0, 2) \\ \sigma _{\beta _{aU}}&\sim {\text {HalfCauchy}}(0, 1) \\ \end{aligned}$$

Finally, the *agency constant model* predicts that agency affects information valuation equally across all levels of probability and uncertainty. This model conceptualised the possibility that the desirability of information is modulated equally by the possession of agency irrespective of the probability of winning and uncertainty of the outcome of the prospect itself. This was achieved by adding the product of a binary parameter, *A*, and an additional free parameter, $\beta _{aC}$, to the existing free parameter $\phi$. The full notation for the *agency constant model* is provided below:

$$\begin{aligned} V_i&\sim {\text {TruncNormal}}(\mu _{V[i]}, \sigma _{V[i]}), \; \in [0, 5] \\ \mu _{V[i]}&= \beta _{U[i]} \, {\bar{H}} + \beta _{W[i]} \, P(W) + (\phi _{i} + \beta _{aC} \, A(t)) \\ \sigma _{V}&\sim {\text {HalfCauchy}}(0, 2) \\ \beta _{W}&\sim {\text {Normal}}(\mu _{\beta _{W}}, \sigma _{\beta _{W}}) \\ \beta _{U}&\sim {\text {Normal}}(\mu _{\beta _{U}}, \sigma _{\beta _{U}}) \\ \phi&\sim {\text {Normal}}(\mu _{\phi }, \sigma _{\phi }) \\ \beta _{aC}&\sim {\text {Normal}}(\mu _{\beta _{aC}}, \sigma _{\beta _{aC}}) \\ \mu _{\beta _{W}}, \mu _{\beta _{U}}, \mu _{\phi }&\sim {\text {Normal}}(0, 2) \\ \mu _{\beta _{aC}}&\sim \text {Normal}(0, 1) \\ \sigma _{\beta _{W}}, \sigma _{\beta _{U}}, \sigma _{\phi }&\sim {\text {HalfCauchy}}(0, 2) \\ \sigma _{\beta _{aC}}&\sim {\text {HalfCauchy}}(0, 1) \\ \end{aligned}$$

## Supplementary information

Supplementary Information.

Supplementary Figure 1.

Supplementary Figure 2.
