# Supplementary material for: Choosing increases the value of non-instrumental information
Source: Sci Rep. 2021 Apr 22;11:8780. doi: 10.1038/s41598-021-88031-y (PMC8062497; doi:10.1038/s41598-021-88031-y)
Supplement: Supplementary file 1 — Supplementary Information. [file 41598_2021_88031_MOESM1_ESM.pdf]

Choosing Increases the Value of Non-Instrumental Information

Matthew Jiwa[1*], Patrick S. Cooper[1,2], Trevor T-J. Chong[2,3,4], and Stefan Bode[1]

[1]University of Melbourne, School of Psychological Sciences, Melbourne, 3010, Australia

[2]Monash University, Turner Institute for Brain and Mental Health, Melbourne, 3800, Australia

[3]Alfred Health, Department of Neurology, Melbourne, 3004, Australia

[4]St Vincent's Hospital, Department of Clinical Neurosciences, Melbourne, 3065, Australia

Supplementary Materials

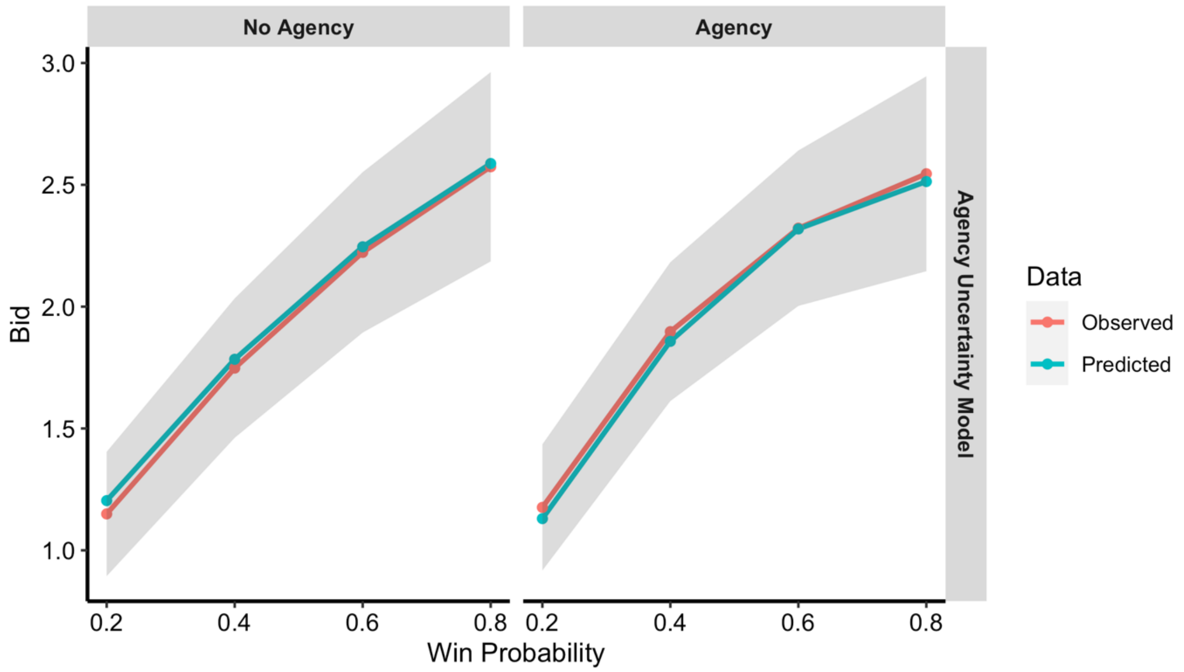

Supplementary Figure S1. Deviance of model predictions from mean bids across win probabilities and agency conditions for the *agency uncertainty model*. The shaded area corresponds to the 95% confidence interval of the observed bids.

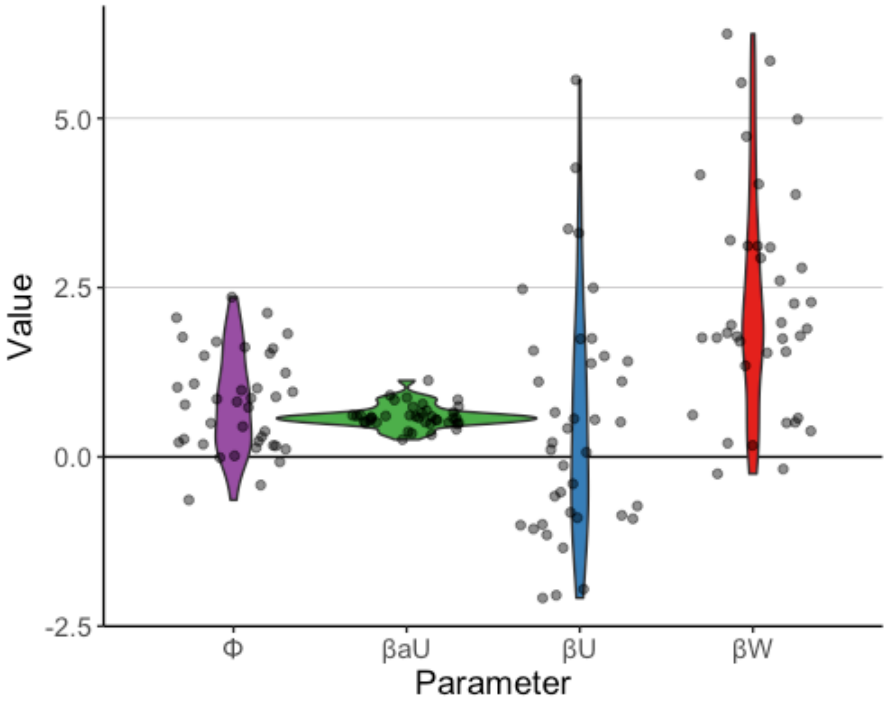

Supplementary Figure S2. Final parameters for the agency uncertainty model. Parameters shown are Φ (the constant value of information), β_{aU}, (the beta parameter for the effect of agency on the value of resolving uncertainty), β_U, (the beta parameter for the value of resolving uncertainty) and β_W (the beta parameter for the value of revealing positive outcomes). Violin plots show the distribution of participants across values for each parameter. Individual participants' parameters are represented by grey dots.
