# Supplementary figures and images for: Choosing increases the value of non-instrumental information
Source: Sci Rep. 2021 Apr 22;11:8780. doi: 10.1038/s41598-021-88031-y (PMC8062497; doi:10.1038/s41598-021-88031-y)

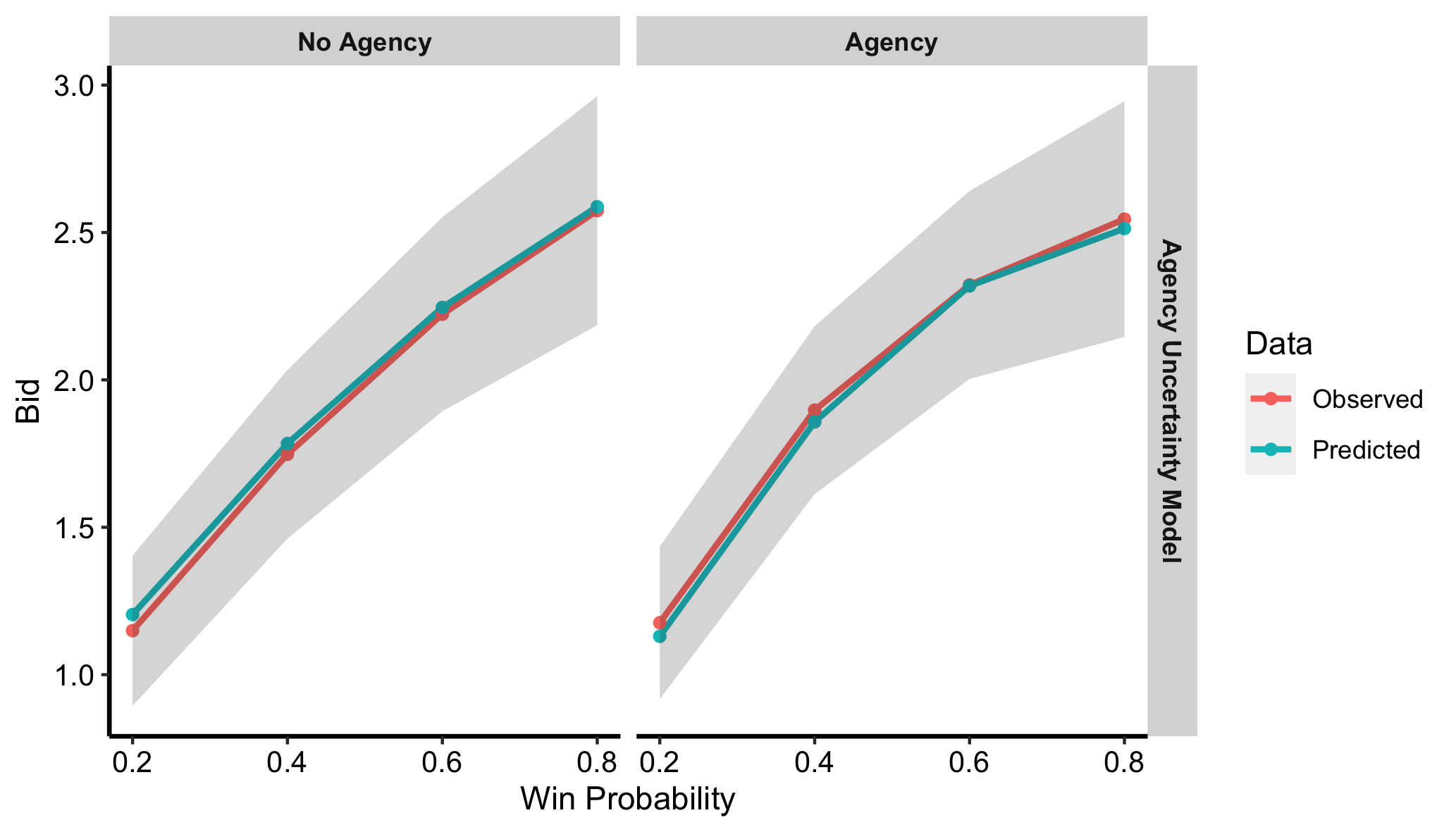

Supplement: Supplementary file 2 — Supplementary Figure 1. [file 41598_2021_88031_MOESM2_ESM.png]

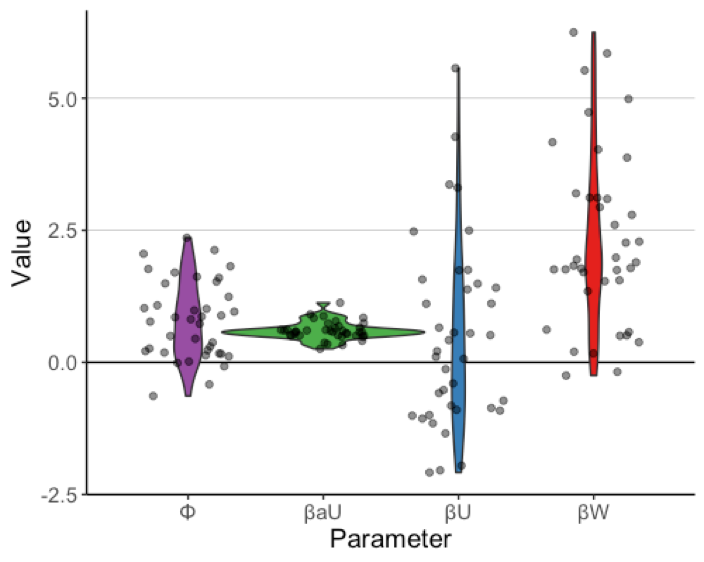

Supplement: Supplementary file 3 — Supplementary Figure 2. [file 41598_2021_88031_MOESM3_ESM.png]
